# ATTITUDES AND PREFERENCES FOR ROBOT-LED PIANO COGNITIVE TRAINING: FEASIBILITY IN MILD COGNITIVE IMPAIRMENT

**DOI:** 10.1093/geroni/igz038.3438

**Published:** 2019-11-08

**Authors:** George Mois, Bailley Collette, Lisa M Renzi-Hammond, Laura Boccanfuso, Aditi Ramachandran, Paul Gibson, Kerstin G Emerson, Jenay M Beer

**Affiliations:** 1 University of Georgia, Athens, Georgia, United States; 2 Institute of Gerontology The University of Georgia, Athens, Georgia, United States; 3 Van Robotics, Columbia, United States; 4 Van Robotics, Columbia, South Carolina, United States; 5 Applied Universal Dynamics, Corp., Loretto, Minnesota, United States

## Abstract

Cognitive training has been shown to improve neural plasticity, increase cognitive reserve and reduce the risk of dementia in older adults. Specifically, learning to play the piano has been shown to be an engaging, multimodal form of cognitive training. However, accessing this form of cognitive training can pose a challenge for older adults. Socially assistive robots present a unique opportunity to increase access to user-tailored piano learning cognitive training. The present study utilized a robot-led four-week piano lesson feasibility intervention for older adults with mild cognitive impairment (N = 11; M= 74.64 ± 6.02 years of age; 72.72% female; 90.1% White/Caucasian). Cognitive Status was assessed during screening via the Telephone Interview for Cognitive Status, and after screening via the Mini-Mental State Exam and the CNS Vital Signs computerized test suite to measure cognitive domain-specific functioning. Perceptions and acceptance of the robot were measured using the Robotic Social Attributes Scale (RoSAS) and Technology Acceptance Scale. Cognitive function improved after four weeks of training in the verbal memory, executive function, reaction time and cognitive flexibility domains, and in the computed neurocognitive index score (p<0.05). Survey data and qualitative interviews show that participants perceived the robot instructor as socially engaging, competent, useful, and easy to use. These results provide insight into the potential of SARs to facilitate cognitive training in the form of piano lessons, as well as recommendations for creating a suitable robot instructor for this application.

